# Artificial intelligence-simplified information to advance reproductive genetic literacy and health equity

**DOI:** 10.1093/humrep/deaf135

**Published:** 2025-07-22

**Authors:** Marjan Naghdi, Ping Cao, Rick Essers, Malou Heijligers, Aimee D C Paulussen, Arie van der Lugt, Robert A C Ruiter, Wendy A G van Zelst-Stams, Andres Salumets, Masoud Zamani Esteki

**Affiliations:** Department of Clinical Genetics, Maastricht University Medical Centre (MUMC+), Maastricht, The Netherlands; Department of Genetics and Cell Biology, GROW Research Institute for Oncology and Reproduction, Maastricht University, Maastricht, The Netherlands; Department of Work and Social Psychology, Faculty of Psychology and Neuroscience, Maastricht University, Maastricht, The Netherlands; Department of Clinical Genetics, Maastricht University Medical Centre (MUMC+), Maastricht, The Netherlands; Department of Genetics and Cell Biology, GROW Research Institute for Oncology and Reproduction, Maastricht University, Maastricht, The Netherlands; Department of Clinical Genetics, Maastricht University Medical Centre (MUMC+), Maastricht, The Netherlands; Department of Genetics and Cell Biology, GROW Research Institute for Oncology and Reproduction, Maastricht University, Maastricht, The Netherlands; Department of Clinical Genetics, Maastricht University Medical Centre (MUMC+), Maastricht, The Netherlands; Department of Clinical Genetics, Maastricht University Medical Centre (MUMC+), Maastricht, The Netherlands; Department of Genetics and Cell Biology, GROW Research Institute for Oncology and Reproduction, Maastricht University, Maastricht, The Netherlands; Section Teaching and Innovation of Learning (STIL), Faculty of Psychology and Neuroscience, Maastricht University, Maastricht, The Netherlands; Department of Work and Social Psychology, Faculty of Psychology and Neuroscience, Maastricht University, Maastricht, The Netherlands; Department of Clinical Genetics, Maastricht University Medical Centre (MUMC+), Maastricht, The Netherlands; Department of Genetics and Cell Biology, GROW Research Institute for Oncology and Reproduction, Maastricht University, Maastricht, The Netherlands; Department of Human Genetics, Radboud Institute for Health Sciences, Radboud University Medical Center, Nijmegen, The Netherlands; Department of Obstetrics and Gynaecology, Institute of Clinical Medicine, University of Tartu, Tartu, Estonia; Division of Obstetrics and Gynaecology, Department of Clinical Science, Intervention & Technology (CLINTEC), Karolinska University Hospital, Karolinska Institutet, Stockholm, Sweden; Celvia CC, Competence Centre on Health Technologies, Tartu, Estonia; Department of Clinical Genetics, Maastricht University Medical Centre (MUMC+), Maastricht, The Netherlands; Department of Genetics and Cell Biology, GROW Research Institute for Oncology and Reproduction, Maastricht University, Maastricht, The Netherlands; Division of Obstetrics and Gynaecology, Department of Clinical Science, Intervention & Technology (CLINTEC), Karolinska University Hospital, Karolinska Institutet, Stockholm, Sweden

**Keywords:** reproductive genetics, artificial intelligence, AI, large language models, LLMs, health literacy, patient education material (PEM), simplification, readability

## Abstract

**STUDY QUESTION:**

Can artificial intelligence (AI) and large language models (LLMs) effectively simplify patient education materials (PEMs) to advance reproductive genetic literacy and health equity?

**SUMMARY ANSWER:**

LLMs offer a promising approach to support healthcare professionals in generating effective, and simplified PEMs.

**WHAT IS KNOWN ALREADY:**

Reproductive genetic testing and counseling holds the potential to support a personalized approach to reduce the burden of genetic disorders. However, its uptake remains limited due to the complexity of the tests and the way that PEMs have been designed. This is more prominent in reproductive genetic testing, as vulnerability of patients may lead to over- or under-use of genetic testing technologies.

**STUDY DESIGN, SIZE, DURATION:**

We carried out a comparative observational study to evaluate the capacity of four AI/LLMs to simplify PEMs (n = 30) in reproductive genetics and assessing the clinical accuracy of simplified versions (n = 120) by experts (n = 30). Additionally, we devised a graphical user interface (GUI) to support real-time text simplification and readability analysis.

**PARTICIPANTS/MATERIALS, SETTING, METHODS:**

We collected 30 PEMs covering six topics in reproductive genetics from well-recognized platforms, such as WHO, MedlinePlus, and Johns Hopkins. Each PEM was processed by four AI/LLMs (GPT-3.5, GPT-4, Copilot, Gemini) using a fixed prompt, resulting in 120 simplified outputs. We measured readability improvements using five validated metrics, such as simple measure of gobbledygook, each capturing distinct textual characteristics such as sentence length and word complexity. To evaluate clinical reliability of the simplified outputs, a panel of experts (n = 30) in reproductive genetics independently scored each text (3 per text).

**MAIN RESULTS AND THE ROLE OF CHANCE:**

All four LLMs significantly improved the readability of the PEMs (*P*-values <0.001), reducing text complexity to an average 6th–7th grade reading level. While Gemini and Copilot achieved the highest improvement in readability scores, GPT-4 received the highest expert rating across all criteria—accuracy (4.1 ± 0.9), completeness (4.2 ± 0.8), and relevance of omissions (4.0 ± 0.9; *P* < 10^−8^). These findings highlight the importance of balancing readability with content integrity to support informed decision-making, as excessive simplification may compromise essential medical information. We devised an open-access GUI that provides real-time PEM simplification and readability analysis to support the integration of AI-assisted approaches in clinical practice (https://huggingface.co/spaces/CellularGenomicMedicine/HealthLiteracyEvaluator).

**LIMITATIONS, REASONS FOR CAUTION:**

Careful evaluation of LLM-simplified PEMs is required to ensure that simplification does not lead to omission of critical information. In addition, in this study, we report only the readability improvements of AI-generated texts and expert evaluations. To truly assess the potential of these tools in advancing reproductive genetic literacy and promoting health equity, real-world patient feedback is essential.

**WIDER IMPLICATIONS OF THE FINDINGS:**

Integrating AI/LLM into patient education strategies may advance health equity by improving understanding and facilitating informed decision-making. Thereby, more effective engagement of patients in reproductive genetic testing programs by assisting them with well-informed decision-making.

**STUDY FUNDING/COMPETING INTEREST(S):**

The EVA specialty program (KP111513) of MUMC+, the Horizon-Europe (NESTOR-101120075), the Estonian Research Council (PRG1076), the Horizon-2020 innovation (ERIN-EU952516) grants of the European Commission, the Swedish Research Council (grant no. 2024-02530), and the Novo Nordisk Foundation (grant no. NNF24OC0092384). The authors declare no conflict of interest relevant to this study.

**TRIAL REGISTRATION NUMBER:**

N/A.

## Introduction

Genomic medicine is revolutionizing healthcare by enabling the integration of genetic data to tailor prevention, diagnosis, and treatment strategies (Porubsky and Eichler, 2024). In this evolving landscape, advanced reproductive genetic testing—including preconception, preimplantation, and prenatal genetic testing—has emerged as a crucial segment of healthcare (Brittain *et al.*, 2017). These tests enable the early detection of genetic abnormalities aiming at preventing the transmission of severe genetic disorders (Capalbo *et al.*, 2021). Despite these rapid advances, about 8 million children worldwide are born each year with birth defects, resulting in substantial childhood morbidity and mortality. The under-use of reproductive genetic testing is particularly acute in low- and middle-income countries, including regions such as South-East Asia, where congenital disorders account for nearly 300 child deaths daily, escalating from 4% to 11% in recent years (WHO, 2024).

Several barriers hinder the widespread and effective use of advanced genetic technologies, including socioeconomic disparities, limited healthcare access, insufficient educating materials in native languages, and notably, low health literacy (van Dijke *et al.*, 2021; Pederson and Narod, 2024). Health literacy, i.e. the capability to obtain, interpret, and understand health information (Weiss, 2003), is influencing both the under- and over-utilization of genetic testing worldwide (Pederson and Narod, 2024). For instance, in the TRIDENT study of the Dutch non-invasive prenatal testing (NIPT) consortium, it became evident that one in three women who declined NIPT lacked sufficient information to make an informed decision (van der Meij *et al.*, 2021). On the other hand, the over-use of preimplantation genetic testing (PGT) for aneuploidies (PGT-A) persists in some clinical settings, despite a lack of robust evidence supporting its effectiveness in its current form (Zamani Esteki *et al.*, 2015; Yan *et al.*, 2021; Janssen *et al.*, 2024), raising the concern about the potential vulnerability of patients who may not fully understand the clinical utility of the test (The Lancet, 2024). The complexity of reproductive genetic information frequently exceeds the comprehension levels of the general population, creating obstacles for patients that can influence their decision-making and the informed consent process (Kay *et al.*, 2025). This, in turn, intensifies existing healthcare disparities and restricts access to potentially life-saving genetic services.

The digital era has transformed patient access to health information, with over 90% of expectant mothers in the USA and Western Europe now relying on online resources for reproductive health guidance (Murphy *et al.*, 2019). This growing trend highlights the pressing need for high-quality, accessible online patient education materials (PEMs). Although the American Medical Association (AMA) and the Centers for Disease Control and Prevention (CDC) recommend that PEMs should be written at or below a 6th- and 8th-grade reading level, respectively (Weiss, 2003; Badarudeen and Sabharwal, 2010), most existing resources remain overly complex, often requiring a college-level understanding (Rooney *et al.*, 2021). This creates a large accessibility gap, marginalizing populations with lower literacy levels, and exacerbating healthcare inequities. Simplifying these PEMs is therefore essential to advancing health equity by ensuring that all individuals, regardless of education, have the knowledge and understanding needed to make informed health decisions. Although health equity, i.e. the right of everybody to achieve optimal health, is primarily influenced by unequal economic, social, and environmental conditions that are not easily changed, enhancing genetic literacy could contribute to improving health equity in the field of reproductive medicine, which is known for its unequal access among families.

Large language models (LLMs) are an emerging type of artificial intelligence (AI) that provides a promising solution for bridging this gap by simplifying complex medical information and making advanced healthcare concepts more accessible to patients and non-experts. ChatGPT (Generative Pre-trained Transformer) is one such model, using transformer-based architectures to generate coherent, human-like text (OpenAI, 2022). LLMs have shown substantial potential across a range of medical applications (Clusmann *et al.*, 2023; Sarangi *et al.*, 2023). Even though the application of AI/LLMs for simplifying information has been explored in certain medical fields (Ayre *et al.*, 2024; Jeblick *et al.*, 2024), to our knowledge, none has (i) investigated their application within the context of reproductive genetics; (ii) engaged an independent expert panel (n = 30) to evaluate the accuracy of LLM-generated PEMs (each text by three experts); (iii) conducted a comparative evaluation of readability improvements across multiple LLMs, targeting recommended reading level between 6th and 8th grade; (iv) analyzed how effectively LLMs optimize textual characteristics essential for accessibility and comprehension; and (v) offered an open access, user-friendly tool to support real-time simplification and readability evaluation of clinical texts.

## Materials and methods

### Study design

**Figure 1.**
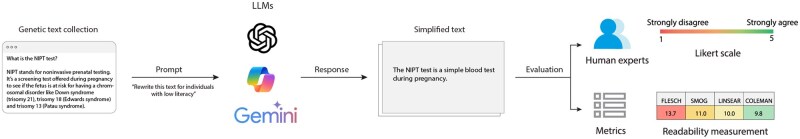
**Study design.** The workflow of the study includes selecting texts from six key reproductive genetic topics, generating simplified outputs using a standardized prompt, and evaluating readability via validated metrics. Accuracy and completeness of the simplified texts were assessed by a panel of human experts (n = 30).

For this study, we devised a comparative observational study to measure the effectiveness of four LLMs, including GPT-3.5, GPT-4, Copilot, and Gemini, in simplifying PEMs on reproductive genetics. The study consisted of three main components: text selection, model-based simplification, and expert evaluation (Fig. 1), which took place between April 2024 and November 2024.

### Data sources and text selection

We sourced 30 original PEMs from reputable healthcare websites, including World Health Organization (WHO), Johns Hopkins Medicine, and MedlinePlus. We based our selection on the following criteria: (i) to ensure a broad range of topics, we included texts on one of six core subjects: reproductive genetic counseling, first-trimester screening, second-trimester screening, amniocentesis, chorionic villus sampling, or NIPT; (ii) to incorporate various writing styles, we chose five PEMs per topic from different websites, creating a dataset of 30 original texts that reflect diverse communication approaches; and (iii) to assess simplicity of the existing PEMs, we focused on texts intended for a general audience, excluding those designed specifically for healthcare professionals.

### Intervention (LLM simplification)

We applied four commonly used LLMs based on their technical features and prior performance in text simplification tasks. GPT-3.5 (OpenAI) served as a widely accessible baseline model, representing an earlier generation of language model technology. GPT-4 (OpenAI), an improved version of GPT-3.5, is known for its advanced reasoning capabilities (OpenAI, 2023). Copilot (Microsoft), previously known as Bing Chat Enterprise, was included due to its strengths in technical and medical content generation (Harper, 2024). Gemini (Google), formerly known as Bard, was chosen for its capabilities in generating structured and clear educational materials, especially in medicine (Saab *et al.*, 2024). We consistently applied a fixed prompt, ‘Rewrite this text for individuals with low literacy’ to all LLMs to safeguard uniformity in task execution. This process generated 120 simplified outputs (30 original texts × 4 LLMs).

### Outcome measures

Our analyses were primarily focused on three domains: (i) readability assessment, (ii) textual characteristics, and (iii) expert evaluations.

### Readability assessment and textual characteristics

To quantify readability, we employed an open-access tool (https://readabilityformulas.com/readability-scoring-system.php) that integrates multiple validated readability metrics, including the Flesch Reading Ease Formula (FRE), Gunning Fog Index (GFI), Coleman–Liau Index (CLI), Linsear Write Formula (LWF), and the Simple Measure of Gobbledygook (SMOG) Index (Supplementary Table S1) (DuBay, 2004). These metrics measure linguistic complexity through various parameters, including sentence length, word length, and the frequency of complex words (Supplementary Data File S1). By using these metrics, we received quantitative readability scores for both the original and LLM-simplified texts that allowed us to make meaningful comparisons of their relative accessibility. To ensure comparability between readability metrics with different scoring ranges (e.g. FRE: 0–100, GFI: 6–17), we applied min–max normalization to rescale each metric to a uniform 0–1 scale, where 0 means the least readable and 1 the most readable. They were then multiplied by 15 to place them on an educational grade-level equivalent scale (Supplementary Table S2), accommodating metrics such as SMOG that extend beyond a 12th-grade reading level.

### Expert evaluation

To evaluate accuracy while avoiding overly precise information, we engaged a panel of 30 experts in reproductive genetics. Following LLM simplification, each text was independently evaluated by three experts through the Qualtrics platform (https://www.qualtrics.com/) (Fisher, 2022). An example of the evaluation survey is provided here: https://qualtricsxmpyt83v8dc.qualtrics.com/jfe/form/SV_eXsqw5QA4b4ySbk. Experts rated texts on three criteria using a 5-point Likert scale: (i) accuracy (fidelity to original content), (ii) completeness (retention of critical information), and (iii) relevance of omissions (ensuring excluded details were unnecessary). To reduce bias, all texts were anonymized, and experts were blind to the LLM source of each output.

### Participants

We recruited an expert panel of 30 professionals specializing in reproductive medicine to evaluate the simplified texts. The inclusion criteria required participants to have formal education in reproductive medicine and a minimum of 5 years of clinical or academic experience. We used professional networks and institutional referrals to recruit participants, ensuring a broad range of perspectives. Specifically, we invited 105 experts, of which 53 started the evaluation. Of these, 30 completed the entire evaluation process.

### Bias mitigation

We implemented five strategies to minimize potential biases. Specifically, we (i) anonymized all LLM outputs to blind the experts (n = 30) to the model that simplified each text, preventing evaluative bias, (ii) consistently applied the same fixed prompt across all LLMs to standardize the simplification process, (iii) randomized text assignments to the experts to avoid order effects and reduce reviewer exhaustion, (iv) sourced each topic from five different websites to mitigate bias arising from specific writing styles, (v) addressed variability in expert evaluations by involving three independent reviewers for each text and averaging their scores to achieve robust assessments.

### Graphical user interface for text simplification and readability evaluation

We developed a graphical user interface (GUI) to facilitate the process of text simplification and readability evaluation. The GUI allows users to upload original texts, and the tool simplifies using LLM. The simplified outputs are then analyzed for readability using the same validated metrics applied in this study, such as the FRE and GFI. These scores are then converted into corresponding grade levels, providing a clear representation of the text complexity. A demo of the GUI is available in Supplementary Fig. S1, and the tool can be accessed via the GitHub repository (https://huggingface.co/spaces/CellularGenomicMedicine/HealthLiteracyEvaluator), providing open access for further research and development.

### Statistical analysis

To assess readability metrics and textual characteristics, we conducted all statistical analyses using R version 4.4.1. To summarize central tendency and variability, we calculated descriptive statistics, including means and SDs. Differences in readability metrics and textual characteristics between the original PEMs and LLM-simplified PEMs were assessed using two-tailed unpaired Student’s *t*-tests, with a significance threshold of 0.05. We then performed a Kruskal–Wallis test to assess variations in expert evaluations across the four LLMs. For visualizing the results, we applied the ggplot2 package in R.

## Results

Our analyses were primarily focused on three domains to assess performance of LLMs in creating simplified information that is precise enough for patients, including (i) readability metrics, (ii) textual characteristics, and (iii) expert evaluations.

### Readability metrics

**Figure 2.**
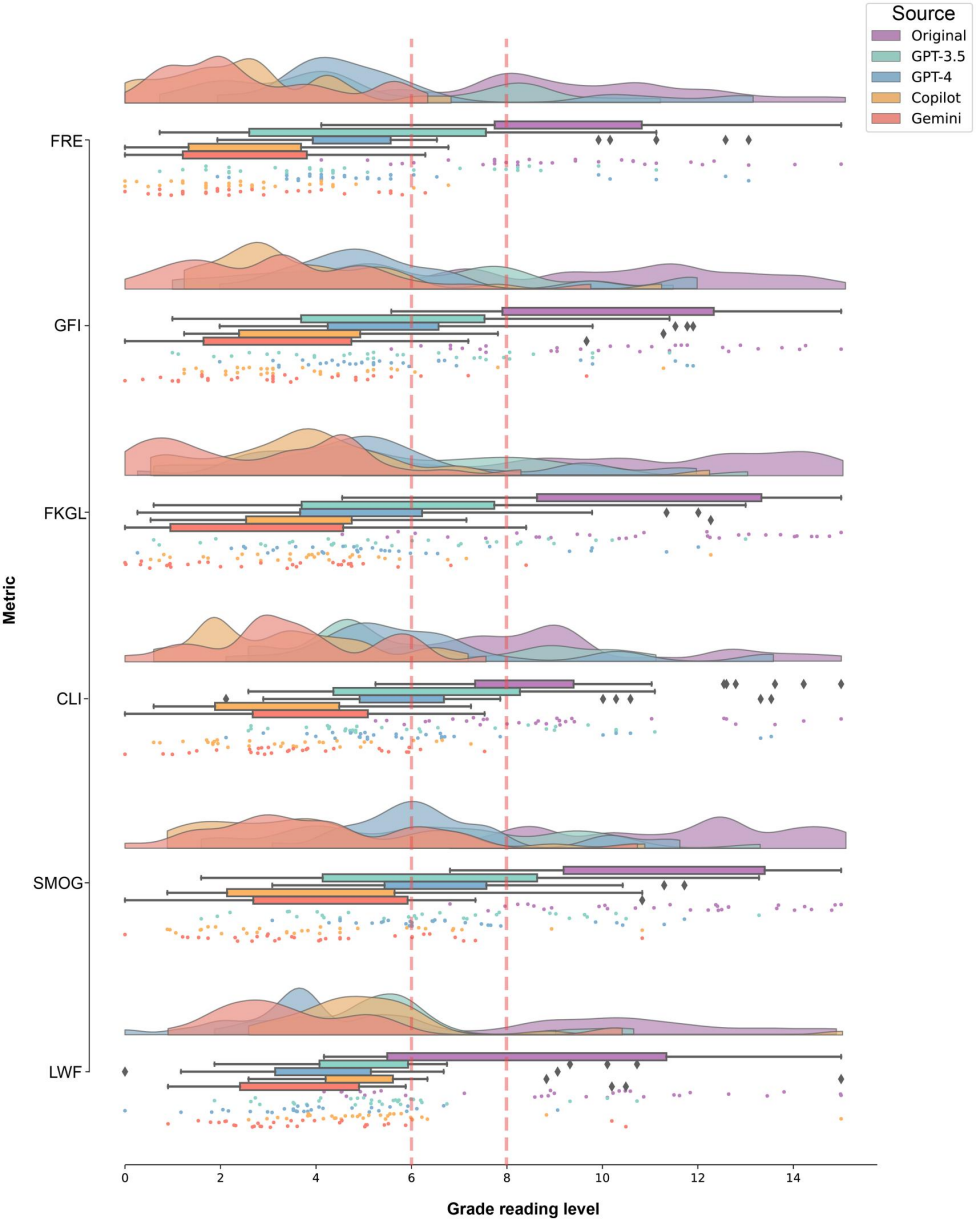
**Comparison of readability metrics.** The original texts (purple curve) show a broad distribution of readability scores, including Flesch Reading Ease (FRE), Gunning Fog Index (GFI), Flesch–Kincaid Grade Level (FKGL), Coleman–Liau Index (CLI), Simple Measure of Gobbledygook (SMOG), and Linsear Write Formula (LWF), indicating high variability and linguistic complexity. The higher scores represent lower readability across all metrics. Gemini and Copilot models have distributions shifted toward higher readability, with narrower peaks reflecting more consistent simplification. GPT-3.5 and GPT-4 also improve readability but with less pronounced effects. Box plots illustrate that original texts have lower median readability scores and larger interquartile ranges (IQRs), indicating greater variability. In contrast, Copilot and Gemini achieve higher median scores and smaller IQRs, signifying more uniform simplification. Scatter plots emphasize readability differences at the individual text level: original texts exhibit numerous outliers representing highly complex samples, whereas Gemini and Copilot models show fewer outliers, indicating more consistent simplification. The red dashed lines at the 6th- and 8th-grade levels indicate the American Medical Association (AMA) and the Centers for Disease Control and Prevention (CDC) recommendations for patient education materials (PEMs) readability, serving as thresholds to highlight the extent of readability improvement.

All four LLMs significantly improved the readability of reproductive genetic texts compared to the original versions (Table 1). The initial assessment of the original PEMs revealed a mean FRE score of 52.0 (±11.0 SD), indicative of an 11th-grade reading level causing a substantial challenge for the public. Copilot and Gemini showed the most pronounced enhancements, with mean FRE scores of 79.5 (±7.1 SD, *P* = 1.5 × 10^−15^) and 79.1 (±7.4 SD, *P* = 2.8 × 10^−15^), respectively. Consistent improvements were observed across all readability metrics, including GFI, FKGL, CLI, SMOG, and LWF, with *P*-values <0.001 (Table 1). The distribution of readability scores demonstrated that all LLMs effectively simplified complex medical texts, with Copilot and Gemini delivering the most accessible outputs (Fig. 2).

**Table 1.** Comparison of readability metrics.

	Original text	GPT-3.5		GPT-4		Copilot		Gemini	
Readability metrics	Mean (SD)	Mean (SD)	***P*-value**	Mean (SD)	***P*-value**	Mean (SD)	***P*-value**	Mean (SD)	***P*-value**
FRE ↑	52.0 (11.0)	69.4 (11.3)	**1.0×10^−7^**	67.1 (11.9)	**4.3×10^−6^**	79.5 (7.1)	**1.5×10^−15^**	79.1 (7.4)	**2.8×10^−15^**
GFI ↓	13.7 (2.1)	10.0 (2.2)	**6.1×10^−9^**	10.1 (2.1)	**1.0×10^−8^**	8.4 (1.7)	**5.0×10^−15^**	8.1 (1.8)	**5.0×10^−16^**
FKGL ↓	10.0 (1.9)	7.0 (1.8)	**3.5×10^−8^**	6.8 (1.7)	**3.4×10^−9^**	5.8 (1.4)	**2.2×10^−13^**	5.3 (1.3)	**1.9×10^−15^**
CLI ↓	11.3 (2.0)	9.0 (1.9)	**3.0×10^−5^**	9.2 (2.1)	**2.3×10^−4^**	7.0 (1.4)	**2.9×10^−13^**	7.1 (1.4)	**8.2×10^−13^**
SMOG ↓	9.9 (1.4)	7.1 (1.6)	**1.8×10^−9^**	7.3 (1.2)	**1.0×10^−10^**	5.7 (1.4)	**6.6×10^−17^**	5.8 (1.3)	**3.4×10^−17^**
LWF ↓	10.6 (2.8)	7.4 (1.7)	**3.3×10^−6^**	6.3 (1.5)	**2.8×10^−9^**	7.3 (1.9)	**2.4×10^−6^**	6.1 (1.9)	**2.3×10^−9^**
Grade level ↓	11.0 (1.6)	8.2 (1.8)	**3.7×10^−8^**	7.9 (1.8)	**1.5×10^−9^**	6.8 (1.5)	**1.4×10^−14^**	6.4 (1.5)	**1.2×10^−16^**

All values presented as mean (±SD). An upward arrow (↑) denotes that higher values correspond to improved performance, while a downward arrow (↓) signifies that lower values indicate better readability. Flesch–Kincaid Grade Level (FRE), Gunning Fog Index (GFI), Coleman–Liau Index (CLI), Linsear Write Formula (LWF), and the Simple Measure of Gobbledygook (SMOG) Index. Bold values indicate statistically significant differences compared to the original text (*P* < 0.001).

### Textual characteristics

To provide a more detailed analysis of how each LLM simplifies PEMs, we evaluated specific textual characteristics, including word count, the percentage of complex words, the prevalence of long sentences, total sentence count, and passive voice usage (Table 2). Text length represented by word count, was higher for the original texts as compared to each individual LLM, with the largest reduction by Gemini (from 450 ± 111.3 SD to 235.2 ± 53.0, *P* = 4.7 × 10^−12^). Lexical complexity represented by percentage of hard words, was higher for the original texts as compared to each individual LLM, with the largest reduction achieved by Copilot (from 19.0% ±4.9 SD to 6.9% ±3.0, *P* = 1.2 × 10^−15^). The original texts contained a higher percentage of long sentences (defined as sentences exceeding 20 words) as compared to each individual LLM, with the largest reduction by GPT-4 (from 26.4% ±17.0 SD to 5.1% ±6.4 SD, *P* = 1.7 × 10^−7^), demonstrating its ability to streamline sentence structure effectively. The total number of sentences was higher for original texts rather than each individual LLM, with the largest reduction by Gemini (from 26.7 ± 8.2 SD to 18.1 ± 4.8 SD, *P* = 9.2 × 10^−6^). The use of passive voice was also significantly reduced by all LLMs, relative to the original texts, with GPT-4 showing the largest reduction (from 11.6% ±6.5 SD to 3.3% ±4.2 SD, *P* = 2.9 × 10^−7^). These findings demonstrate that all LLMs effectively simplified complex medical texts, with significant improvements across all measured variables (*P* < 0.001, two-tailed unpaired Student’s *t*-test), underscoring the ability of LLMs, particularly Gemini, to enhance text readability (Tables 1 and 2, Fig. 2).

**Table 2.** Textual characteristics.

	Original text	GPT-3.5		GPT-4		Copilot		Gemini	
Textual characteristics	Mean (SD)	Mean (SD)	***P*-value**	Mean (SD)	***P*-value**	Mean (SD)	***P*-value**	Mean (SD)	***P*-value**
Word count	450.5 (111.3)	303.1 (70.0)	1.4×10^−7^***	318.3 (76.9)	**2.0×10^−6^** ***	334.8 (84.2)	**3.2×10^−5^** ***	235.2 (53.0)	4.7×10^−12^***
Hard words (%)	19.0 (4.9)	11.7 (5.0)	3.8×10^−7^***	14.4 (5.8)	**1.3×10^−3^** **	6.9 (3.0)	1.2×10^−15^***	8.5 (3.7)	4.7×10^−13^***
Long sentences (>20 words;%)	26.4 (17.0)	13.6 (8.8)	**6.6×10^−4^** **	5.1 (6.4)	**1.7×10^−7^** ***	13.5 (12.5)	**1.5×10^−3^** **	8.3 (12.0)	**1.6×10^−5^** ***
Total sentence	26.7 (8.2)	21.4 (5.5)	**5.1×10^−3^** **	24.3 (6.4)	**2.1×10^−1^**	22.7 (5.7)	**3.6×10^−2^**	18.1 (4.8)	**9.2×10^−6^** ***
Passive voice (%)	11.6 (6.5)	3.7 (4.3)	**1.1×10^−6^** ***	3.3 (4.2)	**2.9×10^−7^** ***	6.0 (5.5)	**6.3×10^−4^** **	3.8 (5.3)	**4.1×10^−6^** ***

Values are presented as mean (SD). Statistical significance was assessed using *t*-tests; *P* < 0.01 (**), *P* < 0.001 (***). Bold values indicate significance differences compared to the original text.

### Expert evaluations

Field experts (n = 30) assessed the accuracy, completeness, and relevance of omissions across all LLM-generated texts (each text by three experts), and a Kruskal–Wallis test was performed for statistical comparisons (Table 3). GPT-4 was overall the highest scoring model, showing highly significant differences in performance compared to all other models. There was a significant difference in accuracy (*P* = 2.8 × 10^−8^), completeness (*P* = 2.1 × 10^−10^), and significance of omissions (*P* = 2.1 × 10^−11^) across LLMs. Moreover, an overall comparison was performed combining all scores from the criteria, showing a significant difference (*P* = 2.2 × 10^−16^) across LLMs. For accuracy, GPT-4 had the highest score and Gemini the lowest (4.1 ± 0.9 and 3.2 ± 1.1, respectively). For completeness, GPT-4 had the highest score and Gemini the lowest (4.2 ± 0.8 and 3.0 ± 1.1, respectively). For significance of omissions, GPT-4 had the highest score and Gemini the lowest (4.0 ± 0.9 and 2.8 ± 1.1, respectively). For the combined score, GPT-4 had the highest score and Gemini the lowest (4.1 ± 0.9 and 3.0 ± 1.1, respectively) (Supplementary Table S3).

**Table 3.** Expert evaluation.

**LLMs** **Criteria**	GPT-3.5	GPT-4	Copilot	Gemini	*P*-value
Accuracy	3.8 (1.1)	4.1 (0.9)	3.5 (1.1)	3.2 (1.1)	2.8×10^−8^
Completeness	3.7 (1.2)	4.2 (0.8)	3.5 (1.1)	3.0 (1.1)	2.1×10^−10^
Relevance of omission	3.5 (1.2)	4.0 (0.9)	3.3 (1.1)	2.8 (1.1)	2.1×10^−11^
All	3.7 (1.1)	4.1 (0.9)	3.6 (1.2)	3.0 (1.1)	2.2×10^−16^

Mean (SD) scores for criteria across the four large language models (a Likert score of 1 represents the worst rating, while a score of 5 represents the best rating). Statistical significance was determined through Kruskal–Wallis test. Reported *P*-values indicate the statistical significance of differences between models. LLMs, large language models.

**Figure 3.**
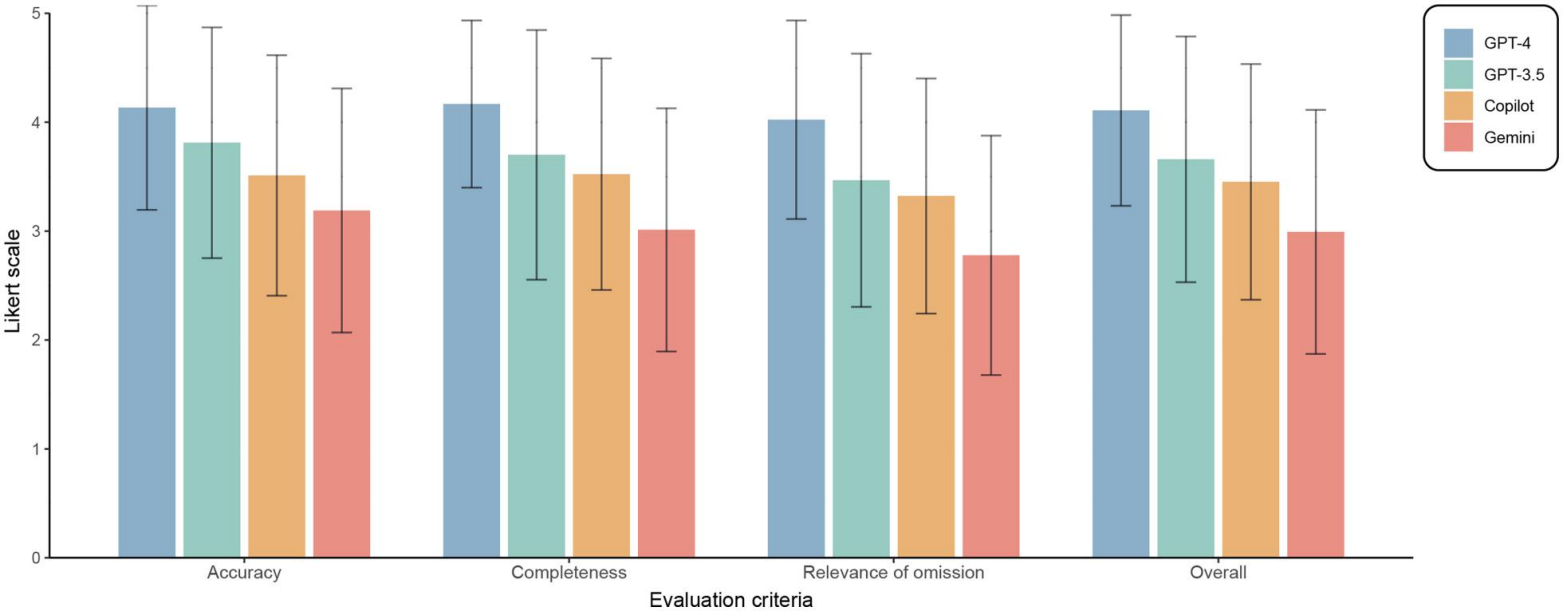
**Expert Evaluation of large language models (LLMs).** Expert assessment of four (LLMs) across three evaluation criteria, including accuracy, completeness, and significance of omissions, as well as overall performance. Scores are provided on a 5-point Likert scale, where higher values indicate better performance. Error bars represent the SD, reflecting the variability in expert assessments.

Although Gemini effectively simplified texts based on the readability metrics, maintaining essential content is compromised with lower expert evaluation scores (Table 4). In contrast, GPT-4 outperformed all other models across evaluation metrics, with consistently high scores and minimal variability, as reflected in its shorter error bars (Fig. 3). These findings emphasize that while all LLMs improve text accessibility, GPT-4 strikes the best balance between simplification and preserving essential information, making it the most reliable model for patient education purposes.

**Table 4.** Comparison of original and simplified descriptions of first-trimester screening.

Original detail	Simplified version	Missing/changed information
Nuchal translucency screening using **transvaginal ultrasound**	A picture of the baby’s neck checks for extra fluid	No mention of NT or transvaginal ultrasound
Measures **PAPP-A** and **hCG** produced by the **placenta**	Checks for ‘special things’ in your blood	Technical names removed, source of substances missing.
Screens for **Down syndrome (trisomy 21)** and **trisomy 18**	May show ‘an increased risk of certain birth defects’	No specific conditions mentioned
Can lead to **additional testing** if results are abnormal	‘Might need further tests’	No examples of tests like **amniocentesis** or **cell-free DNA testing**
Risk of **false positives/false negatives** explained in detail	Briefly mentioned	Less clear about the consequences and why additional testing is important

The table presents side-by-side examples of original patient education content and their simplified versions generated by large language models (LLMs), along with notes on missing or altered information in the simplified texts.

## Discussion

Health equity requires not only equitable access to healthcare services but also ensuring that individuals are adequately informed about their healthcare options. This principle is particularly critical in reproductive health, where insufficient awareness can place patients at a disadvantage situation (The Lancet, 2024), potentially resulting in missed opportunities for timely and essential interventions. Receiving adequate reproductive care can also prevent future guilt feelings due to poorly founded choices. Furthermore, a lack of awareness may lead to unnecessary treatments and procedures, exacerbating concerns that the fertility industry exploits patient vulnerability for profit (The Lancet, 2024). By enhancing the availability of reproductive health information, we can address this vulnerability, empower patients with knowledge, protect their autonomy, and facilitate informed decision-making.

Our analysis of existing PEMs from reputable sources revealed considerable challenges in achieving these goals. For instance, the technical complexity and highly specialized nature of materials related to different forms of PGT made them unsuitable as general patient education resources. To avoid potential bias in our results, these materials were excluded from our study. Moreover, PGT is provided only to a specific group of patients, including mostly infertile couples, and not for all pregnant women. Yet, this exclusion highlights a huge gap in truly patient-centered resources for advanced reproductive technologies. Here, we demonstrated that LLMs could meaningfully increase PEM readability, aligning with findings from prior research across diverse healthcare domains (Clusmann *et al.*, 2023; Sarangi *et al.*, 2023; Ayre *et al.*, 2024). We found that Copilot and Gemini showed the most substantial improvements, reducing the reading level of materials to a 6th- to 7th-grade level, which aligns with the recommendations from the AMA and CDC for accessible health materials. These results indicate that LLMs hold promise for improving the accessibility of PEMs, particularly for patients with low literacy, supporting informed decision-making in reproductive genetics. However, our results also revealed a critical trade-off between readability and content integrity. While Copilot and Gemini excelled in simplifying text, they often omitted important medical details, which could compromise patient understanding and decision-making. This challenge is consistent with prior evaluations of LLMs in preventive medicine and primary care, where models sometimes excluded key details despite producing mostly accurate responses (Kassab *et al.*, 2024). In contrast, GPT-4 offered a more balanced performance, maintaining higher levels of accuracy and completeness while still improving readability. It also became evident that GPT-4 had a better performance as compared to GPT-3.5. These findings suggest that advanced LLMs with enhanced reasoning capabilities may be better suited for tasks requiring a careful balance between simplicity and accuracy. Our concerns regarding the risk of oversimplification at the cost of accuracy align with broader concerns about the limitations of LLMs in healthcare, including risks such as ‘AI hallucinations’ (de Hond *et al.*, 2024; Ong *et al.*, 2024; The Lancet Digital Health, 2024), where models produce incorrect or misleading information, and ‘automation bias’ (Chen *et al.*, 2024), where clinicians may over-rely on AI-generated content without sufficient scrutiny. For instance, a recent evaluation of ChatGPT’s responses to urology case scenarios found that only 52% of its recommendations were deemed appropriate by experts, raising concerns about the reliability of LLMs in handling complex or time-sensitive medical information (Cocci *et al.*, 2024). These risks highlight the critical need for human oversight in reviewing LLM-simplified texts prior to dissemination.

While our study focused on patient understanding, simplifying complex medical information also has important implications for healthcare providers. In reproductive medicine, where informed consent is increasingly complex, LLM-generated content could help providers better grasp and communicate key concepts. Our findings align with recent applications of generative AI in simplifying consent forms and clinical texts summarization (Mirza *et al.*, 2024; Van Veen *et al.*, 2024), suggesting that such tools may enhance understanding between patients and healthcare professionals. Future studies that measure intra-annotation influences, i.e. bias evaluation of the same expert in different time points, would benefit these studies by reducing the temporary subjective view and enhancing the expert opinion on a specific topic. Additionally, while it is possible that some of the original PEMs used in our study were included in the pretraining datasets of the LLMs, especially those sourced from widely available public websites such as MedlinePlus or WHO, the exact training data of proprietary models like GPT-4 or Gemini are not publicly disclosed. Importantly, to reduce potential familiarity bias, we included PEMs from multiple sources and ensured diverse topics and writing styles.

Unlike conventional manual approaches or prior studies that mainly focused on readability metrics, our GUI integrates the entire process, text simplification, and readability assessment into a single, streamlined platform. This integrated tool optimizes the efficiency and consistency of readability assessment, delivering immediate feedback to support the real-time refinement of PEMs. It also enhances accessibility for researchers and clinicians dealing with health information materials. However, further research is needed to examine how these simplified materials are received by patients in real-world clinical settings, assessing their impact on comprehension, decision-making, and health outcomes. Moreover, the LLMs used in the current study are largely restricted to English language speakers and PEMs. Their use in other languages may be hindered by the need to translate the PEM into English and then back into the original language after LLM-based text simplification. Since we showed improvement of GPT from 3.5 to 4, it is likely that evolution of a certain LLM with more data and optimized training can improve AI-assisted PEMs simplification with more accurate information. Importantly, ongoing evaluation, supported by active participation from healthcare professionals and patients, is necessary to refine LLM-simplified PEMs and facilitate their safe and effective integration into clinical practice.

## Conclusion

The main aim of technological advancement is to benefit humans, and this study demonstrates the potential of LLMs in healthcare particularly in making complex medical information easier to understand. Using these capabilities to increase patient understanding, especially among those with low literacy, is a valuable achievement in promoting health equity. However, it is important that this simplification does not result in oversimplification or misinformation. Therefore, human oversight remains critical for protecting the reliability and clarity of health information prepared by AI.

## Supplementary Material

deaf135_Supplementary_Data_File_S1

deaf135_Supplementary_Figure_S1

deaf135_Supplementary_Table_S1

deaf135_Supplementary_Table_S2

deaf135_Supplementary_Table_S3

## Data Availability

Anonymized data, including original texts, LLM-generated outputs, and detailed analysis source codes are available at (https://github.com/CellularGenomicMedicine/HealthLiteracyEvaluator).
