# Supplementary material for: Artificial intelligence-simplified information to advance reproductive genetic literacy and health equity
Source: Hum Reprod. 2025 Jul 22;40(9):1681–8. doi: 10.1093/humrep/deaf135 (PMC12408898; doi:10.1093/humrep/deaf135)
Supplement: deaf135_Supplementary_Figure_S1 [file deaf135_supplementary_figure_s1.pdf]

OXFORD
human reproduction
Human Reproduction, 2025, 1–1
https://doi.org/10.1093/humrep/deaf135
Supplementary Data

A Initial interface layout

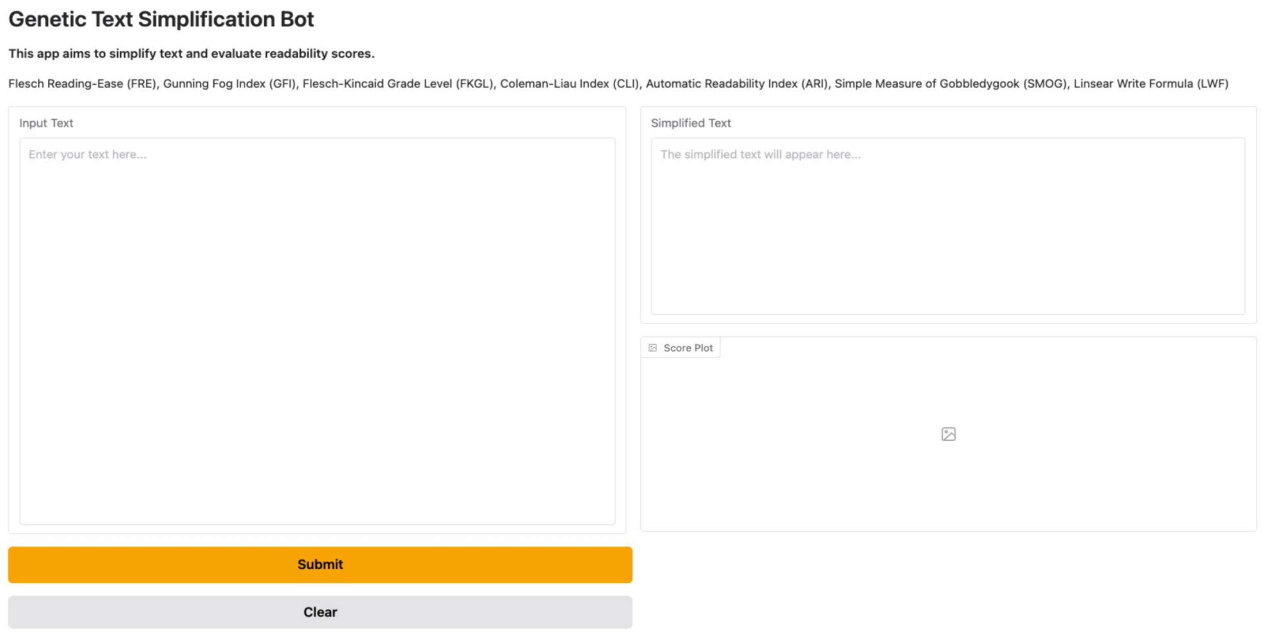

B Simplified text output and readability scores

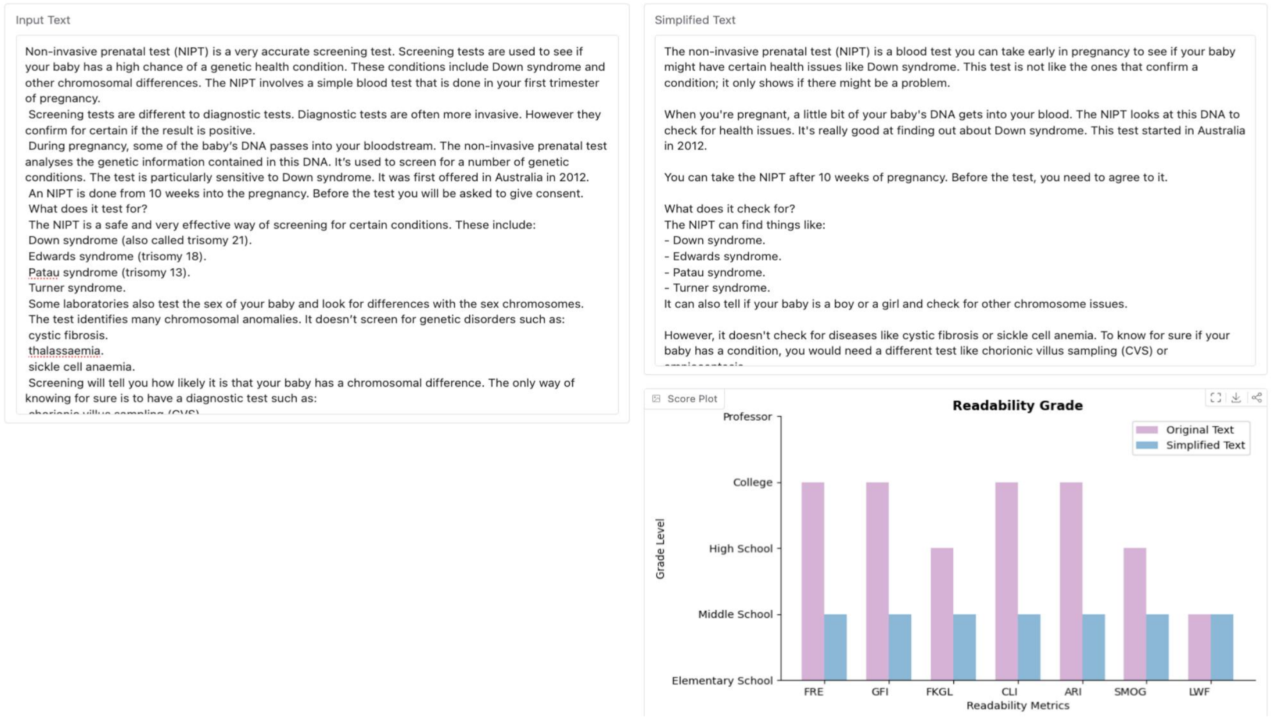

Supplementary Figure S1. Graphical user interface (GUI) for text simplification and readability evaluation. (**A**) Depicts the initial interface layout, allowing users to input or upload original texts for simplification. (**B**) Illustrates the output interface, displaying the simplified text alongside a visual comparison of readability metrics.
