# Supplementary material for: Artificial intelligence-simplified information to advance reproductive genetic literacy and health equity
Source: Hum Reprod. 2025 Jul 22;40(9):1681–8. doi: 10.1093/humrep/deaf135 (PMC12408898; doi:10.1093/humrep/deaf135)
Supplement: deaf135_Supplementary_Table_S1 [file deaf135_supplementary_table_s1.pdf]

Human Reproduction, 2025, 1–1
https://doi.org/10.1093/humrep/deaf135
Supplementary Data

Supplementary Table S1. Readability metrics.

Test name	Measures	Formula	Score range	Interpretation
Flesch Reading Ease Formula (FRE)[1]	• Sentence length<br>• Word length<br>• Higher scores easier readability	$FRE = 206.835 - 1.015\left(\frac{\text{total words}}{\text{total sentences}}\right) - 84.6\left(\frac{\text{total syllables}}{\text{total words}}\right)$	0–100	• 90–100: Very easy<br>• 80–89: Easy<br>• 70–79: Fairly easy<br>• 60–69: Standard<br>• 50–59: Fairly difficult<br>• 30–49: Difficult<br>• 0–29: Very difficult
Gunning Fog Index (GFI)[2]	• Sentence length<br>• Complex words<br>• Lower scores, easier readability	$GFI = 0.4 \times \left[\left(\frac{\text{total words}}{\text{total sentences}}\right) + 100\left(\frac{\text{total complex words}}{\text{total words}}\right)\right]$	6–17	• 6–9: 6th–9th grade<br>• 9–12: High school<br>• 13–15: College<br>• 16–17: Professional
Flesch–Kincaid Grade Level (FKGL)[3]	• Sentence length<br>• Word length<br>• Lower scores, easier readability	$FKGL = 0.39\left(\frac{\text{total words}}{\text{total sentences}}\right) + 11.8\left(\frac{\text{total syllables}}{\text{total words}}\right) - 15.59$	0–12	• 0–5.9: Elementary school<br>• 6–8.9: Middle school<br>• 9–12.9: High school<br>• 13+: College
Coleman–Liau Index (CLI)[4]	• Character per word<br>• Sentence length<br>• Lower scores, easier readability	$CLI = 5.89\left(\frac{\text{total characters}}{\text{total words}}\right) - 0.3\left(\frac{\text{total sentences}}{\text{total words}}\right) - 15.8$	1–12	• 1–6: Elementary school<br>• 7–8: Middle school<br>• 9–10: High school<br>• 11–12: College
Simplified Measure of Gobbledegook Index (SMOG)[5]	• Polysyllabic words<br>• Sentence count<br>• Lower scores, easier readability	$SMOG = 1.043\sqrt{\text{total polysyllable words} \times \frac{30}{\text{total sentences}}} + 3.1291$	0–18	• 0–6: Elementary school<br>• 7–9: Middle school<br>• 10–12: High school<br>• 13–18: College
Linsear Write Formula (LWF)[6]	• Designed for technical text<br>• Words syllables<br>• Lower scores, easier readability	$LWF = \frac{\left[\left(100 - \left(\frac{100 \times n_{wsy<3}}{n_w}\right)\right) + \left(3 \times \frac{100 \times n_{wsy>=3}}{n_w}\right)\right]}{\left(100 \times \frac{n_{st}}{n_w}\right)}$<br>$\boldsymbol{nwsy < 3}$ = the number of words with less than 3 syllables, and $\boldsymbol{nwsy \geq 3}$ = the number of words with 3 syllables or more.	1–12	• 1–5: Elementary school<br>• 6–8: Middle school<br>• 9–10: High school<br>• 11–12: College

1. Kincaid JP, Fishburne RP Jr, Rogers RL, Chissom BS. *Derivation of New Readability Formulas (Automated Readability Index, Fog Count and Flesch Reading Ease Formula) for Navy Enlisted Personnel*. Institute for Simulation and Training. 1975;56.
2. Gunning R. *The Technique of Clear Writing*. New York: McGraw-Hill; 1952.
3. Kincaid JP. *Development and Test of a Computer Readability Editing System (CRES)*. Final Report, June 1978 through December 1979. Naval Training Analysis and Evaluation Group, 1980.
4. Coleman M, Liau TL. A computer readability formula designed for machine scoring. *J Appl Psychol* 1975;**60**:283–284. [CrossRef][10.1037/h0076540][InsertedFromOnline]
5. Mc Laughlin GH. SMOG grading—a new readability formula. *J Read* 1969;**12**:639–646.
6. O'Hayre J. *Gobbledygook Has Gotta Go*. Washington (DC): US Department of the Interior, Bureau of Land Management; 1966.
