# Supplementary material for: Artificial intelligence-simplified information to advance reproductive genetic literacy and health equity
Source: Hum Reprod. 2025 Jul 22;40(9):1681–8. doi: 10.1093/humrep/deaf135 (PMC12408898; doi:10.1093/humrep/deaf135)
Supplement: deaf135_Supplementary_Table_S2 [file deaf135_supplementary_table_s2.pdf]

OXFORD
human reproduction

Human Reproduction, 2025, 1–1
https://doi.org/10.1093/humrep/deaf135
Supplementary Data

Supplementary Table S2. Educational grade levels.

Age	US/International grades	Educational level
6–7	1st Grade	**Elementary school**
7–8	2nd Grade	
8–9	3rd Grade	
9–10	4th Grade	
10–11	5th Grade	
11–12	6th Grade	**Middle school**
12–13	7th Grade	
13–14	8th Grade	
14–15	9th Grade	**High school**
15–16	10th Grade	
16–17	11th Grade	
17–18	12th Grade	
