# Supplementary material for: Artificial intelligence-simplified information to advance reproductive genetic literacy and health equity
Source: Hum Reprod. 2025 Jul 22;40(9):1681–8. doi: 10.1093/humrep/deaf135 (PMC12408898; doi:10.1093/humrep/deaf135)
Supplement: deaf135_Supplementary_Table_S3 [file deaf135_supplementary_table_s3.pdf]

OXFORD
human reproduction

*Human Reproduction*, 2025, 1–1
https://doi.org/10.1093/humrep/deaf135
Supplementary Data

**Supplementary Table S3.** Individual expert ratings and assessment of inter-annotator variability across large language models (LLMs).

| Expert No. | Accuracy GPT-3.5 | Completeness GPT-3.5 | Omissions GPT-3.5 | Accuracy Copilot | Completeness Copilot | Omissions Copilot | Accuracy Gemini | Completeness Gemini | Omissions Gemini | Accuracy GPT-4 | Completeness GPT-4 | Omissions GPT-4 |
|---|---|---|---|---|---|---|---|---|---|---|---|---|
| **Exp 1** | 4 | 5 | 5 | 4 | 4 | 4 | 3 | 3 | 3 | 5 | 5 | 5 |
| **Exp 2** | 5 | 4 | 4 | 4 | 2 | 3 | 3 | 2 | 3 | 5 | 4 | 4 |
| **Exp 3** | 4 | 2 | 2 | 4 | 2 | 2 | 2 | 2 | 2 | 5 | 5 | 5 |
| **Exp 4** | 4 | 5 | 4 | 3 | 4 | 2 | 3 | 3 | 2 | 4 | 4 | 4 |
| **Exp 5** | 4 | 4 | 4 | 2 | 2 | 2 | 5 | 5 | 5 | 2 | 3 | 3 |
| **Exp 6** | 4 | 5 | 4 | 4 | 4 | 4 | 2 | 3 | 2 | 4 | 3 | 4 |
| **Exp 7** | 5 | 5 | 4 | 4 | 4 | 4 | 4 | 4 | 4 | 5 | 5 | 5 |
| **Exp 8** | 4 | 4 | 4 | 2 | 2 | 2 | 5 | 5 | 5 | 2 | 3 | 3 |
| **Exp 9** | 5 | 5 | 5 | 1 | 2 | 1 | 4 | 4 | 4 | 5 | 5 | 5 |
| **Exp 10** | 5 | 5 | 5 | 2 | 3 | 3 | 4 | 4 | 4 | 5 | 5 | 5 |
| **Exp 11** | 4 | 2 | 4 | 4 | 4 | 2 | 4 | 4 | 3 | 5 | 4 | 4 |
| **Exp 12** | 4 | 4 | 4 | 4 | 2 | 2 | 4 | 4 | 2 | 4 | 4 | 4 |
| **Exp 13** | 3 | 4 | 4 | 3 | 3 | 3 | 3 | 3 | 3 | 2 | 3 | 4 |
| **Exp 14** | 4 | 3 | 2 | 3 | 4 | 3 | 3 | 2 | 3 | 4 | 4 | 3 |
| **Exp 15** | 5 | 4 | 5 | 4 | 3 | 4 | 3 | 3 | 2 | 4 | 4 | 4 |
| **Exp 16** | 5 | 5 | 5 | 4 | 4 | 4 | 3 | 3 | 3 | 5 | 5 | 5 |
| **Exp 17** | 3 | 3 | 3 | 3 | 2 | 2 | 3 | 2 | 3 | 5 | 4 | 4 |
| **Exp 18** | 4 | 5 | 5 | 4 | 5 | 5 | 3 | 3 | 3 | 5 | 4 | 5 |
| **Exp 19** | 4 | 4 | 4 | 5 | 5 | 5 | 5 | 4 | 5 | 5 | 5 | 5 |
| **Exp 20** | 2 | 2 | 2 | 2 | 2 | 2 | 2 | 2 | 2 | 4 | 4 | 4 |
| **Exp 21** | 5 | 5 | 5 | 5 | 5 | 5 | 3 | 3 | 3 | 4 | 4 | 4 |
| **Exp 22** | 4 | 4 | 3 | 3 | 3 | 3 | 4 | 4 | 4 | 2 | 3 | 2 |
| **Exp 23** | 2 | 2 | 2 | 2 | 2 | 2 | 1 | 1 | 1 | 4 | 4 | 4 |
| **Exp 23** | 2 | 2 | 2 | 3 | 4 | 3 | 3 | 2 | 2 | 4 | 4 | 4 |
| **Exp 24** | 4 | 4 | 3 | 4 | 4 | 4 | 4 | 4 | 4 | 4 | 3 | 4 |
| **Exp 25** | 5 | 5 | 4 | 2 | 2 | 2 | 2 | 1 | 1 | 4 | 4 | 3 |
| **Exp 26** | 2 | 3 | 4 | 4 | 3 | 3 | 4 | 4 | 4 | 3 | 4 | 3 |
| **Exp 27** | 4 | 4 | 3 | 3 | 3 | 2 | 3 | 2 | 3 | 4 | 4 | 4 |
| **Exp 28** | 5 | 5 | 5 | 4 | 3 | 3 | 2 | 2 | 2 | 4 | 4 | 4 |
| **Exp 29** | 2 | 2 | 2 | 3 | 3 | 2 | 3 | 3 | 2 | 4 | 4 | 4 |
| **Exp 30** | 3 | 3 | 3 | 4 | 4 | 4 | 3 | 3 | 2 | 5 | 5 | 4 |
| **Exp 1** | 4 | 4 | 2 | 5 | 5 | 5 | 4 | 4 | 3 | 5 | 5 | 5 |
| **Exp 2** | 5 | 4 | 4 | 5 | 4 | 4 | 4 | 4 | 3 | 4 | 3 | 2 |
| **Exp 3** | 5 | 4 | 4 | 4 | 4 | 4 | 2 | 2 | 2 | 5 | 5 | 5 |
| **Exp 4** | 4 | 4 | 4 | 4 | 4 | 4 | 5 | 5 | 5 | 5 | 5 | 5 |
| **Exp 5** | 2 | 4 | 4 | 2 | 4 | 4 | 1 | 2 | 2 | 4 | 4 | 4 |
| **Exp 6** | 4 | 4 | 4 | 2 | 2 | 2 | 2 | 2 | 2 | 2 | 4 | 4 |
| **Exp 7** | 4 | 4 | 4 | 4 | 4 | 4 | 5 | 5 | 5 | 5 | 5 | 5 |
| **Exp 8** | 2 | 2 | 1 | 1 | 4 | 2 | 1 | 1 | 1 | 5 | 5 | 5 |
| **Exp 9** | 2 | 2 | 2 | 2 | 2 | 2 | 4 | 4 | 4 | 2 | 2 | 2 |
| **Exp 10** | 5 | 4 | 4 | 3 | 3 | 3 | 1 | 1 | 1 | 5 | 5 | 5 |
| **Exp 11** | 5 | 5 | 4 | 4 | 4 | 4 | 3 | 3 | 2 | 5 | 5 | 5 |
| **Exp 12** | 2 | 2 | 2 | 3 | 3 | 3 | 3 | 2 | 3 | 3 | 4 | 3 |
| **Exp 13** | 5 | 5 | 5 | 3 | 3 | 3 | 2 | 2 | 2 | 4 | 4 | 4 |
| **Exp 14** | 4 | 4 | 5 | 3 | 3 | 2 | 3 | 2 | 2 | 3 | 3 | 2 |
| **Exp 15** | 5 | 5 | 3 | 2 | 4 | 3 | 3 | 3 | 4 | 5 | 4 | 3 |
| **Exp 16** | 2 | 3 | 2 | 5 | 5 | 5 | 3 | 4 | 2 | 5 | 5 | 5 |
| **Exp 17** | 4 | 4 | 4 | 4 | 2 | 2 | 4 | 4 | 4 | 4 | 4 | 4 |
| **Exp 18** | 5 | 4 | 4 | 4 | 3 | 3 | 3 | 3 | 3 | 5 | 5 | 5 |
| **Exp 19** | 5 | 4 | 4 | 5 | 5 | 5 | 4 | 4 | 3 | 5 | 5 | 5 |
| **Exp 20** | 5 | 5 | 5 | 4 | 2 | 2 | 3 | 2 | 2 | 3 | 4 | 4 |
| **Exp 21** | 4 | 2 | 2 | 5 | 5 | 5 | 4 | 3 | 2 | 4 | 5 | 5 |
| **Exp 22** | 4 | 4 | 3 | 5 | 5 | 5 | 5 | 5 | 2 | 5 | 5 | 5 |
| **Exp 23** | 2 | 2 | 2 | 2 | 4 | 4 | 4 | 4 | 4 | 2 | 2 | 2 |
| **Exp 23** | 2 | 2 | 2 | 4 | 4 | 4 | 3 | 3 | 3 | 4 | 4 | 4 |
| **Exp 24** | 2 | 3 | 2 | 2 | 2 | 2 | 4 | 4 | 3 | 5 | 4 | 4 |
| **Exp 25** | 4 | 1 | 2 | 5 | 5 | 5 | 5 | 5 | 5 | 5 | 5 | 5 |
| **Exp 26** | 4 | 5 | 3 | 2 | 2 | 2 | 2 | 2 | 2 | 2 | 3 | 4 |
| **Exp 27** | 3 | 2 | 2 | 4 | 4 | 4 | 3 | 3 | 3 | 3 | 3 | 2 |
| **Exp 28** | 5 | 5 | 3 | 4 | 5 | 3 | 5 | 5 | 3 | 5 | 5 | 3 |
| **Exp 29** | 4 | 4 | 4 | 4 | 4 | 4 | 5 | 4 | 4 | 5 | 5 | 5 |
| **Exp 30** | 4 | 3 | 2 | 3 | 4 | 2 | 4 | 4 | 2 | 5 | 5 | 5 |

(continued)

OXFORD

human reproduction

*Human Reproduction*, 2025, 1–1
https://doi.org/10.1093/humrep/deaf135
Supplementary Data

**Supplementary Table S3.** (continued)

| Expert No. | Accuracy GPT-3.5 | Completeness GPT-3.5 | Omissions GPT-3.5 | Accuracy Copilot | Completeness Copilot | Omissions Copilot | Accuracy Gemini | Completeness Gemini | Omissions Gemini | Accuracy GPT-4 | Completeness GPT-4 | Omissions GPT-4 |
|---|---|---|---|---|---|---|---|---|---|---|---|---|
| **Exp 1** | 4 | 3 | 3 | 4 | 4 | 4 | 2 | 2 | 2 | 5 | 4 | 5 |
| **Exp 2** | 5 | 5 | 5 | 4 | 4 | 4 | 4 | 3 | 3 | 5 | 5 | 5 |
| **Exp 3** | 4 | 4 | 4 | 4 | 4 | 4 | 3 | 3 | 3 | 4 | 4 | 4 |
| **Exp 4** | 4 | 4 | 4 | 4 | 4 | 4 | 3 | 2 | 2 | 5 | 5 | 5 |
| **Exp 5** | 4 | 5 | 4 | 5 | 5 | 5 | 2 | 2 | 1 | 4 | 5 | 5 |
| **Exp 6** | 4 | 5 | 2 | 4 | 3 | 3 | 1 | 2 | 1 | 4 | 3 | 2 |
| **Exp 7** | 2 | 4 | 2 | 4 | 4 | 4 | 4 | 5 | 5 | 4 | 4 | 4 |
| **Exp 8** | 2 | 2 | 2 | 4 | 4 | 4 | 1 | 1 | 1 | 4 | 4 | 4 |
| **Exp 9** | 3 | 2 | 2 | 4 | 5 | 4 | 4 | 4 | 2 | 5 | 5 | 5 |
| **Exp 10** | 4 | 5 | 5 | 4 | 4 | 4 | 1 | 1 | 1 | 4 | 4 | 4 |
| **Exp 11** | 5 | 4 | 5 | 5 | 4 | 5 | 4 | 4 | 2 | 4 | 3 | 2 |
| **Exp 12** | 4 | 4 | 5 | 3 | 2 | 2 | 2 | 2 | 2 | 4 | 4 | 4 |
| **Exp 13** | 4 | 4 | 4 | 3 | 4 | 3 | 2 | 2 | 2 | 4 | 4 | 4 |
| **Exp 14** | 5 | 5 | 5 | 5 | 5 | 5 | 4 | 4 | 4 | 5 | 4 | 4 |
| **Exp 15** | 4 | 4 | 4 | 4 | 3 | 3 | 2 | 2 | 2 | 4 | 4 | 4 |
| **Exp 16** | 4 | 4 | 4 | 4 | 4 | 2 | 2 | 2 | 2 | 5 | 5 | 5 |
| **Exp 17** | 4 | 2 | 1 | 5 | 5 | 5 | 4 | 4 | 4 | 4 | 5 | 5 |
| **Exp 18** | 4 | 4 | 4 | 4 | 4 | 4 | 4 | 2 | 2 | 4 | 4 | 4 |
| **Exp 19** | 5 | 5 | 5 | 5 | 5 | 5 | 4 | 3 | 3 | 5 | 5 | 5 |
| **Exp 20** | 4 | 4 | 4 | 4 | 4 | 4 | 3 | 3 | 3 | 3 | 3 | 3 |
| **Exp 21** | 2 | 2 | 2 | 2 | 2 | 2 | 4 | 3 | 2 | 4 | 4 | 4 |
| **Exp 22** | 4 | 4 | 2 | 5 | 5 | 5 | 4 | 4 | 5 | 5 | 5 | 5 |
| **Exp 23** | 2 | 2 | 2 | 2 | 4 | 2 | 2 | 2 | 2 | 2 | 4 | 4 |
| **Exp 23** | 2 | 2 | 2 | 2 | 2 | 2 | 4 | 3 | 2 | 5 | 5 | 5 |
| **Exp 24** | 2 | 2 | 2 | 2 | 2 | 3 | 2 | 2 | 2 | 4 | 4 | 4 |
| **Exp 25** | 5 | 5 | 5 | 2 | 2 | 3 | 2 | 2 | 2 | 5 | 5 | 4 |
| **Exp 26** | 3 | 2 | 2 | 5 | 5 | 3 | 4 | 4 | 4 | 3 | 4 | 3 |
| **Exp 27** | 4 | 3 | 4 | 3 | 3 | 3 | 4 | 2 | 2 | 4 | 4 | 4 |
| **Exp 28** | 4 | 4 | 4 | 1 | 2 | 2 | 2 | 3 | 3 | 4 | 3 | 3 |
| **Exp 29** | 5 | 5 | 4 | 5 | 5 | 3 | 5 | 5 | 3 | 5 | 5 | 3 |
| **Exp 30** | 4 | 4 | 4 | 4 | 4 | 4 | 3 | 3 | 3 | 4 | 5 | 4 |
| **Mean** | **3.81** | **3.7** | **3.47** | **3.51** | **3.52** | **3.32** | **3.2** | **3.01** | **2.78** | **4.1** | **4.2** | **4.02** |
| **SD** | **1.1** | **1.2** | **1.12** | **1.1** | **1.1** | **1.1** | **1.12** | **1.12** | **1.1** | **0.94** | **0.8** | **0.91** |

In this table, we present the scores given by each expert (n = 30) for all LLM-simplified texts, evaluated across three criteria: accuracy, completeness, and relevance of omissions. Each text was rated independently by three experts. To reflect inter-annotator variability, we included the mean and SD of scores at the bottom of the table for each LLM and criterion. Bold values indicate the mean score per criterion across models, including their associated standard deviation. The standard deviation reflects the level of agreement between expert ratings, with lower values indicating greater consistency.

© The Author(s) 2025. Published by Oxford University Press on behalf of European Society of Human Reproduction and Embryology.
This is an Open Access article distributed under the terms of the Creative Commons Attribution License (https://creativecommons.org/licenses/by/4.0/), which permits unrestricted reuse, distribution, and reproduction in any medium, provided the original work is properly cited.
Human Reproduction, 2025, 00, 1–13
https://doi.org/10.1093/humrep/deaf135
Original Article
